# Incidence and risk factors for postpartum hemorrhage in Uganda

**DOI:** 10.1186/s12978-016-0154-8

**Published:** 2016-04-14

**Authors:** Sam Ononge, Florence Mirembe, Julius Wandabwa, Oona M. R. Campbell

**Affiliations:** Department of Obstetrics and Gynaecology, Makerere University College of Health Sciences, P.O. Box 7072, Kampala, Uganda; Department of Obstetrics and Gynaecology, Busitema University Faculty of Health Sciences, Mbale, Uganda; Faculty of Epidemiology and Population Health, London School of Hygiene and Tropical Medicine, London, UK

**Keywords:** Labor, Oxytocics, Pregnancy, Postpartum hemorrhage, Risk factors

## Abstract

**Background:**

Globally, postpartum haemorrhage (PPH) remains a leading cause of maternal deaths. However in many low and middle income countries, there is scarcity of information on magnitude of and risk factors for PPH (blood loss of 500 ml or more). It is important to understand the relative contributions of different risk factors for PPH. We assessed the incidence of, and risk factors for postpartum hemorrhage among rural women in Uganda.

**Methods:**

Between March 2013 and March 2014, a prospective cohort study was conducted at six health facilities in Uganda. Women were administered a questionnaire to ascertain risk factors for postpartum hemorrhage, defined as a blood loss of 500 mls or more, and assessed using a calibrated under-buttocks drape at childbirth. We constructed two separate multivariable logistic regression models for the variables associated with PPH. Model 1 included all deliveries (vaginal and cesarean sections). Model 2 analysis was restricted to vaginal deliveries. In both models, we adjusted for clustering at facility level.

**Results:**

Among the 1188 women, the overall incidence of postpartum hemorrhage was 9.0 %, (95 % confidence interval [CI]: 7.5–10.6 %) and of severe postpartum hemorrhage (1000 mls or more) was 1.2 %, (95 % CI 0.6–2.0 %). Most (1157 [97.4 %]) women received a uterotonic after childbirth for postpartum hemorrhage prophylaxis. Risk factors for postpartum hemorrhage among all deliveries (model 1) were: cesarean section delivery (adjusted odds ratio [aOR] 7.54; 95 % CI 4.11–13.81); multiple pregnancy (aOR 2.26; 95 % CI 0.58–8.79); foetal macrosomia ≥4000 g (aOR 2.18; 95 % CI 1.11–4.29); and HIV positive sero-status (aOR 1.93; 95 % CI 1.06–3.50). Risk factors among vaginal deliveries only, were similar in direction and magnitude as in model 1, namely: multiple pregnancy, (aOR 7.66; 95 % CI 1.81–32.34); macrosomia, (aOR 2.14; 95 % CI1.02–4.47); and HIV positive sero-status (aOR 2.26; 95 % CI 1.20–4.25).

**Conclusion:**

The incidence of postpartum hemorrhage was high in our setting despite use of uterotonics. The risk factors identified could be addressed by extra vigilance during labour and preparedness for PPH management in all women giving birth.

## Background

Globally, postpartum hemorrhage (PPH) is a leading cause of maternal mortality [1]. The global prevalence of PPH is 6 % [2] and the highest burden is experienced in low-income countries [3, 4]. The magnitude of PPH in sub-Saharan Africa is high at 10.5 % [2]. In Uganda, PPH causes 25 % of all maternal deaths [5]. However, there is little information on the magnitude and risk factors for PPH. Common causes of PPH are uterine atony, genital tract injuries, failure of the blood coagulation system and trauma. Uterine atony is responsible for the majority (75 %) of PPH [6]. Risk factors for PPH include; past history of PPH, multiple pregnancy, fetal macrosomia, primi-gravida, grand multi-parity, older age, preterm births, genital tract injuries, non-use of oxytocics for PPH prophylaxis, labour induction, cesarean birth and intra-uterine fetal deaths [4, 7–10]. However, majority of these studies visually estimated blood loss, a method that has considerable inaccuracy [11]. The few studies that have objectively measured blood loss after childbirth are in high-income countries, whose delivery settings differ from those in low income countries [12]. This study used a calibrated under-buttocks drape to measure blood loss that is highly correlated with the gold-standard measure of photospectometry [13]. Knowledge of the risk factors would inform public health interventions for PPH control. To the clinicians, risk factor identification in the antenatal and intrapartum periods may provide an opportunity for timely interventions to prevent PPH. The present study was undertaken to objectively assess the incidence and risk factors for PPH among women in rural health facilities in Uganda.

## Methods

Uganda’s health system has 7 facility levels. As part of a stepped-wedge cluster-randomized trial on prevention of PPH using misoprostol (Registered in Pan African Clinical Trials Network: PACTR201303000459148), a prospective cohort study was conducted among women who delivered at six lower level health facilities (5 health centre IIIs and one health centre IV) in Mpigi district, Central Uganda between 6th March 2013 and 19th March 2014 [14]. The health centre IIIs, serve a sub-county with average population of 25,000 people [15], and are staffed by clinical officers, nurses, midwives, laboratory technician, and a health inspector. Maternity services offered at health centre III are basic essential obstetric care. In addition, these health facilities offer prevention of mother to child transmission of Human Immune deficiency virus (HIV) services which encompasses routine counseling and testing of all women attending the antenatal clinic. Those found HIV positive are routinely offered highly active anti-retroviral therapy provided by government of Uganda [16]. Medical high risk and complicated obstetric cases from health centre IIIs are referred to either health centre IVs or to hospitals (levels V–VII). Health centre IVs serve a health sub-district with average population of 95,000. They are staffed as for Health centre III with the addition of a medical officer(s) and an anaesthetic officer. Health centre IVs provide comprehensive essential obstetric care, including caesarean section [17]. The six health centers in the study were purposively selected out of 9 eligible health facilities according to the trial inclusion criteria. Ethical approval was obtained from the School of Medicine Research and Ethics Committee at Makerere University, Kampala, Uganda, and the Uganda National Council for Science and Technology. Informed written consent was obtained from all participants at enrolment.

### Data collection

**Fig. 1:**
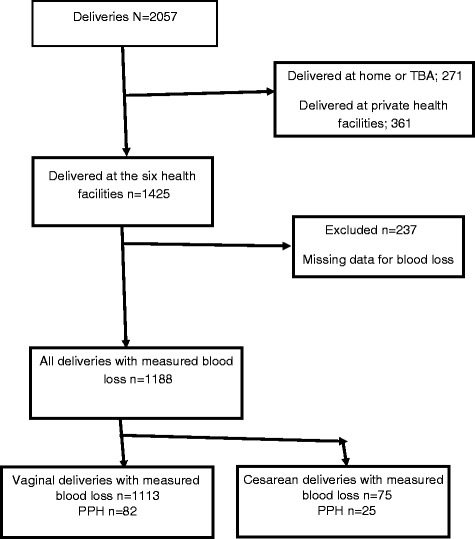
Profile of the study participants

Pregnant women were recruited at 28 weeks gestation or greater. The trial included women who planned to stay in the district during pregnancy, delivery and in the immediate postpartum period. The study excluded women who had a planned elective caesarean-section delivery or who had previous caesarean-section scars. The participants who delivered at the six health facilities were included in this study. Women who had missing data for the primary outcome of blood loss (16.6 %) were excluded in the analysis (Fig. 1). The health providers in the delivery rooms in these health facilities were trained on the data collection procedure and on measurement of postpartum blood loss. During enrolment, interviewer-administered questionnaires were used to collect data on the risk factors including: previous history of PPH, woman’s age in completed years, parity and HIV sero-status obtained from patient records. Participants’ hemoglobin levels were measured using a potable hemoCue^R^ Hb 301 system at recruitment. Gestational age at birth was calculated based on ultrasound scan estimation, the self-report of last normal menstrual period (LNMP), or fundal height estimation. The research team noted whether labour was induced or augmented with oxytocin, the mode of delivery, performance of episiotomy, perineal tear requiring suture, single or multiple deliveries, use of oxytocics at birth to prevent PPH (injectable oxytocin 10 IU or oral misoprostol 600 micrograms) within 1 min of delivery. Birth weight was measured using a Seca Medical 725 mechanical baby scale. The primary outcome was postpartum hemorrhage defined as blood loss of 500 mls or more after childbirth, while severe postpartum hemorrhage was a blood loss of 1000 mls or more. In women who had vaginal birth, postpartum blood loss was measured using a calibrated under-buttocks V-BRASSS drape [13]. After delivery of the baby and clamping of the umbilical cord, a drape was placed under the woman’s buttocks. Blood was allowed to flow into the drape for an hour or until the attending midwife felt that the flow of blood is inconsequential. The total blood loss collected in the calibrated conical receptacle was established by the attending midwife. The used drapes with their contents were disposed of correctly by the attending midwife. In women who had cesarean section, we relied on the visual estimation of blood loss by the operating clinician.

### Statistical analysis

Data were double entered in Epidata 3.1, cleaned and exported to STATA 12 for analysis. Descriptive statistics provided the average blood loss and incidence of PPH. The incidence of PPH in the first hour (immediate) postpartum was calculated as total number of women with blood loss of 500 ml or more, divided by the total study sample and, was presented as a percentage. Characteristics that were continuous and categorical variables were calculated as means and proportions respectively. Bivariate logistic regression analysis of the risk factors for PPH was done to estimate crude odds ratios (cORs) and their 95 % confidence interval. We constructed two separate multivariable logistic regression models for the variables associated with PPH. Model 1 included all deliveries (vaginal and cesarean sections). Model 2 analysis was restricted to only vaginal deliveries. Factors that had a p-value less than 0.2 at bivariate analysis were entered into multivariable logistic regression analysis to estimate the adjusted odds ratios (aORs). In model 1, covariates adjusted for in multivariable logistic regression included parity, maternal anaemia, past history of PPH, HIV sero-status, mode of delivery, multiple pregnancy, macrosomia (4000 g or more) and perineal tears. In model 2, mode of delivery was omitted. Because the participants in the study were from 6 clusters (health facilities), we adjusted for clustering in the logistic regression. A statistical significance was defined as a p value <0.05.

We estimated that with prevalence of PPH of 6 %, .a sample size of 1171 women would give a 90 % power to detect a 40 % increased risk for PPH in grand-multiparous women [2].

## Results

Data collected among 1188 women showed mean blood loss of 236 ml (±193 ml) and ranged from 50 to 1800 mls. Overall, 107 (9.0 %, 95 %; CI 7.5–10.6 %) women had PPH and 14 (1.2 %, 95 %; CI 0.6–2.0 %) had severe PPH (1000 mls or more). Table 1 shows the distribution of socio-demographic, antepartum and intrapartum characteristics of the study participants, and their association with PPH. In general women with PPH tended to be older, more likely to have twin births and HIV infection. They were also more likely to have delivered a macrosomic baby, be postdates or have received no uterotonic. Overall, only 31 (2.6 %) women did not receive a uterotonic at childbirth for PPH prevention. Main reasons for not receiving uterotonic at birth were facility stock outs of drugs and syringes. Among those who did receive uterotonics, oxytocin was the most frequent drug administered. Out of the 82 (6.9 %) women who received additional uterotonics at birth, majority (78) got misoprostol. Women who had PPH were more than twice more likely to have received additional uterotonic than those who did not have PPH (14.0 % vs 6.2 %). The prevalence of PPH and severe PPH among the 1113 women with vaginal deliveries were 7.4 % and 0.7 % respectively. Seven (0.6 %) women received blood transfusion after childbirth, while 6 (0.5 %) were referred to hospital because of lack of blood products at the study facility.

**Table 1:** Association between the risk factors and postpartum hemorrhage among all deliveries

Variable	No PPH (*n* = 1,081)^a^	Had PPH (*n* = 107)^a^	Crude OR (95 % CI)	*P* value	Adjusted OR (95 % CI)	*P* value
Parity						
Primiparous	301 (27.8)	31 (29.0)	0.97 (0.61–1.53)	0.47	0.85 (0.49–1.44)	0.30
2–4	545 (50.4)	58 (54.2)	Reference		Reference	
5+	235 (21.7)	18 (16.8)	0.72 (0.42–1.25)		0.64 (0.34–1.15)	
Previous history of PPH	47 (4.4)	9 (8.4)	2.02 (0.96–4.24)	0.06	1.86 (0.81–4.26)	0.16
Gestation age at birth (weeks)						
Preterm (<37)	181 (16.7)	16 (15.0)	0.90 (0.51–1.58)	0.76	0.80 (0.44–1.46)	0.71
Term (37–41)	762 (70.5)	75 (70.1)	Reference		Reference	
Post term (>41)	138 (12.8)	16 (15.0)	1.18 (0.67–2.08)		1.07 (0.58–1.99)	
Multiple pregnancy	10 (0.9)	4 (3.7)	4.16 (1.28–13.50)	0.02	2.26 (0.58–8.79)	0.26
Moderate-severe anemia in pregnancy	121 (11.2)	15 (14.0)	1.14 (0.85–1.52)	0.38	0.99 (0.72–1.35)	0.97
HIV positive	98 (9.1)	17 (15.9)	1.89 (1.08–3.31)	0.03	1.93 (1.06–3.50)	0.04
Cesarean-section birth	50 (4.6)	25 (23.4)	6.29 (3.70–10.68)	<0.001	7.54 (4.11–13.81)	<0.001
Labour induction/augmented	42 (3.9)	3 (2.8)	0.71 (0.22–2.34)	0.58		
Macrosomia ≥4000gm	78(7.2)	15(14.0)	2.10(1.16–3.79)	0.01	2.25(1.14–4.43)	0.03
No uterotonic at birth	25 (2.3)	6 (5.6)	2.51(1.01–6.26)	0.05	2.23 (0.80–6.21)	0.14
Additional uterotonic	67 (6.2)	15 (14.0)				
Episiotomy/Perineal tear	133 (12.3)	13 (12.1)	0.99 (0.54–1.81)	0.96	1.44 (0.71–2.94)	0.32

*Abbreviation: PPH* Postpartum hemorrhage, *OR* odds ratio, *CI* confidence interval, *HIV* Human Immunodeficiency Virus

^a^ Values are given as numbers (percentages)

In model 1 that included both the vaginal and cesarean deliveries Table 1, factors associated with increased risk for PPH included: HIV sero positivity (aOR 1.93; 95 % CI 1.06–3.50); cesarean section (aOR 7.54; 95 % CI 4.11–13.81); and fetal macrosomia ≥4000 g (aOR 2.18; 95 % CI 1.11–4.29). Multiple pregnancy and no uterotonic after childbirth, though significant at unadjusted analysis, were not associated with PPH after controlling for other risk factors. In the second model (only vaginal deliveries) Table 2, factors associated with increased risk for PPH at logistic regression analysis included: HIV sero positivity (aOR 2.26; 95 % CI 1.20–4.25); multiple pregnancy (aOR 7.66; 95 % CI 1.81–32.34); and fetal macrosomia ≥4000 g (aOR 2.14; 95 % CI 1.02–4.47). On the other hand, grand multi-parity was marginally associated with PPH (*p* = 0.07).

**Table 2:** Association between the risk factors and postpartum hemorrhage in vaginal deliveries^a^

Variable	No PPH *n* = 1,031 (%)	PPH ≥500 mls *n* = 82 (%)	Crude OR (95 % CI)	*P* value	Adjusted OR (95 % CI)	*P* value
Age in years				0.89		
≤19	199 (19.3)	17 (20.7)	1.11 (0.63–1.95)			
20–34	754 (73.1)	58 (70.7)	Ref			
>34	78 (7.6)	7 (8.5)	1.17 (0.51–2.64)			
Mean age (standard deviation)	24.5 (5.7)	24.4 (6.0)		0.93		
Married	902 (87.5)	68 (82.9)	0.69 (0.38–1.27)	0.24		
Grand multi-Parity	230 (22.3)	14 (17.1)	0.72 (0.40–1.30)	0.27	0.57 (0.31–1.08)	0.07
Past history of PPH	45 (4.4)	9 (11.0)	2.7 (1.27–5.74)	0.01	2.08 (0.89–4.85)	0.11
Gestational age at birth				0.82		0.84
Preterm (<37 weeks)	170 (16.5)	12 (14.6)	0.89 (0.47–1.69)		0.85 (0.43–1.67)	
Term (37–41 weeks)	730 (70.8)	58 (70.7)	Reference		Reference	
Post term (>41 weeks)	131 (12.7)	12 (14.6)	1.15 (0.60–2.21)		1.08 (0.54–2.16)	
Multiple pregnancy	6 (0.6)	4 (4.9)	8.76 (2.42–31.7)	0.001	7.66 (1.81–32.34)	0.01
Anaemia in pregnancy				0.58		0.84
No anaemia	696 (67.5)	53 (64.6)	Reference		Reference	
Mild anaemia	225 (21.8)	17 (20.7)	1.06 (0.64–1.75)		0.97 (0.58–1.62)	
Moderate-severe anaemia	110 (10.7)	12 (14.6)	1.72 (0.65–4.57)		1.37 (0.47–3.98)	
HIV positive	92 (8.9)	15 (18.3)	2.29 (1.25–4.16)	0.007	2.26 (1.20–4.25)	0.02
Macrosomia ≥4000 g	75 (7.3)	10 (12.2)	1.77 (0.88–3.57)	0.87	2.14 (1.02–4.47)	0.04
Uterotonic after birth				0.02		0.31
None	25 (2.4)	6 (7.3)	4.12 (1.22–13.8)		2.80 (0.75–10.4)	
Oxytocin	903 (87.6)	70 (85.4)	Reference		Reference	
Misoprostol	103 (10)	6 (7.3)	1.32 (0.56–3.13)		1.36 (0.56–3.27)	
Additional uterotonic	67 (6.5)	14 (17.1)	2.96 (1.58–5.54)	0.001		
Labor induced/ augmented with oxytocin	37 (3.6)	1 (1.2)	0.33 (0.04–2.45)	0.28		
Episiotomy/Perineal tear	131 (12.7)	13 (15.9)	1.29 (0.70–2.41)	0.42	1.44 (0.73–2.83)	0.29

*Abbreviation: CI* confidence interval, *HIV* Human Immunodeficiency Virus, *PPH* Postpartum hemorrhage, *OR* odds ratio; ^a^ Values are given as numbers (percentages)

All 14 (1.2 %) participants who developed severe PPH received fluid replacement. Five women were given additional oxytocin, two received blood transfusion and two were transferred from health centre III to hospital; unfortunately one died en-route. Of all participants who developed severe PPH, six were delivered by cesarean section, and one was among those transfused. One woman had cervical tear that was successfully repaired in theatre under general anesthesia.

## Discussion

This study demonstrated that, almost all (97 %) women delivering at the six health facilities in rural Uganda received a uterotonic for prevention of PPH. The overall incidence of PPH was 9.0 % and of severe PPH was 1.2 %. The risk factors for PPH were being HIV positive, multiple pregnancy, delivery by cesarean section and delivering a macrosomic baby. The study minimized classification bias of PPH by objectively measuring the blood loss after child-birth using a calibrated drape.

The incidence of PPH in this study is higher than the reported global rate of 6 % by Carroli et al [2], however similar incidence of 10.8 % was reported by Calvert et al [12]. This high incidence of PPH in this study may have been influenced by the characteristics of the study population; these were rural women that are reported to have higher rates of PPH. Studies that have compared urban and rural women have reported higher rates of PPH in the later [2, 18]. The high rates in rural areas could be related to substandard care. It is also possible that the use of calibrated drape in assessment of postpartum bleeding could have influenced the apparent high rate of PPH since assessment of postpartum blood loss using a calibrated drape is more objective way of determining the rate of PPH. Most studies that report lower rates of PPH have used visual estimation of blood loss [4] which is known to underestimate the amount of measured blood loss by 30 % [11]. Nevertheless since this study found that the vast majority of women received oxytocin, it is somewhat surprising that PPH was so high. This raises concern about the cold chain maintenance for the storage of oxytocin in rural settings where electricity is not readily available. A national survey done in 2007 on active management of the third stage of labour found that 34.3 % of health facilities were storing oxytocin at room temperature [19]. Using an alternative uterotonic such as misoprostol that can be stored at room temperature would be an option to consider in setting with unstable electricity supply. However, we noted that in our study, women getting misoprostol versus oxytocin for prophylaxis had a 36 % increased odds of PPH. Delivery by cesarean section was associated with increased risk of PPH. This is consistent with previous studies that report cesarean births being associated with increased risk of PPH [4, 6, 20]. However, few studies report a protective effect of cesarean section against PPH when compared to vaginal births [21, 22]. The lower rates of PPH in cesarean section than in vaginal birth are in studies that included participants not in labor. In our study, the cesarean sections were performed in women who were in labor and where the operative mode of delivery was decided by attending physician for the good of the unborn baby and woman, with some of the cesarean sections probably being done late in advanced stages of labour. There is increased risk of severe PPH when cesarean sections are performed when the cervical dilatation is more than 9 cm or in second stage of labor mainly due to avulsion of the blood vessels at the delivery of impacted presenting part [23]. Our finding support existing evidence showing that cesarean section increases the risk of PPH [2, 4, 6, 20, 24]. Cesarean section also increases risk of uterine atony, a leading cause of PPH [6, 25]. Because women undergoing cesarean section are at increased risk of PPH, health units performing them should have blood bank capacity to respond to a need for blood transfusion when required. In addition, its important for clinicians to re-assess the women destined for emergency cesarean section when near or in second stage of labour for possible instrumental vaginal (vacuum extraction or forceps) delivery. These modes of delivery have been observed to reduce the rates of cesarean section [26].

The risk of PPH was doubled in large (macrosomia, ≥4000 g) babies. The increased risk of PPH among women giving birth to large babies has also been documented in previous studies [8, 9]. Large babies are known to over-distend the uterus which is associated with uterine atony. This findings call for more vigilance on the part of practitioners attending labor and births to identify women at risk, have adequate preparation and plan for early intervention to prevent PPH. Multiple pregnancy was associated with increased risk of PPH. This is consistent with previous studies that report multiple pregnancy being associated with PPH [6, 10, 20, 25, 27]. The over distension caused by multiple pregnancy increases the risk of uterine atony. In addition, the large placental size in multiple increases the surface area for bleeding after childbirth.

Our findings showed that HIV positive pregnant women were more likely to have PPH than HIV negative, even in the analysis among vaginal births alone. Though a review of literature show that there is no significant difference in PPH rates between HIV positive and negative women [28], one subsequent study showed an increased risk of severe PPH among HIV positive women [29]. While the mechanism through which HIV causes PPH is not yet known, some have suggested that the increased thrombocytopenia that is observed in 10–30 % of HIV positive persons might be the one responsible for hemorrhage [30, 31]. Alternatively, it has been suggested that low level of care offered to HIV positive women at birth [28] could be responsible for the increased risk, whereby the health providers might be hesitant to implement an intervention when bleeding starts for fear of exposing themselves especially when there is no adequate protective gear. Our findings add to the growing body of knowledge, highlighting the impact of HIV on the health of women. This has clinical implications for the care of HIV positive pregnant in setting with high prevalence of HIV. There is a need for more research to further elucidate the problem and establish the mechanisms whereby HIV may lead to PPH.

This study had the following limitations. First, the findings are from health facilities and almost all women received a uterotonic. It is known in Uganda that 48 % of deliveries occur at home [32]. Thus, the findings may not be generalizable to home deliveries. However, with exception of one study site (Health Centre IV), the study was conducted in lower level health facilities where services like blood transfusion and surgery (cesarean section) are not offered. Secondly, women who delivered by cesarean section had blood measured by visual estimation that has been known to underestimate blood loss. However, these were 75 (6 %), and are unlikely to have substantially affected the estimate of PPH risk. Thirdly there were only 14 cases with severe PPH. The study did not have adequate power to identify risk factors for severe PPH.

## Conclusion

The incidence of PPH was high despite many women receiving uterotonic at delivery of the babies. The risk factors for PPH in our setting were cesarean section, multiple pregnancy, fetal macrosomia and HIV. Extra vigilance during the antenatal and peripartum periods is needed to identify women at risk and early intervention to prevent PPH. It is important to remember that we have to prepare for PPH in all women giving birth, as some get PPH without any known risk factors.

## Abbreviations

Hb: Haemoglobin
HC: Health Centre
HIV: Human Immune-deficiency Virus
LNMP: Last Normal Menstrual Period
PPH: Postpartum Haemorrhage
WHO: World Health Organisation
